# Chemoprevention of neuroblastoma: progress and promise beyond uncertainties

**DOI:** 10.20517/2394-4722.2022.40

**Published:** 2023-03-31

**Authors:** Natarajan Aravindan, Mohan Natarajan, Dinesh Babu Somasundaram, Sheeja Aravindan

**Affiliations:** 1 Department of Radiation Oncology, University of Oklahoma Health Sciences Center, Oklahoma City, OK 73104, USA.; 2 Stephenson Cancer Center, Oklahoma City, OK 73104, USA.; 3 Department of Pathology and Laboratory Medicine, University of Texas Health Sciences Center at San Antonio, San Antonio, TX 78229, USA.

**Keywords:** Neuroblastoma, chemoprevention, secondary and tertiary chemopreventive, phytochemicals, retinoids, polyphenols, terpenes, DFMO, apoptosis

## Abstract

Neuroblastoma is the most common extracranial solid tumor in children and comprises one-tenth of all childhood cancer deaths. The current clinical therapy for this deadly disease is multimodal, involving an *induction phase* with alternating regimens of high-dose chemotherapeutic drugs and load reduction surgery; a *consolidation phase* with more intensive chemotherapy, radiotherapy, and stem cell transplant; and a *maintenance phase* with immunotherapy and immune-activating cytokine treatment. Despite such intensive treatment, children with neuroblastoma have unacceptable life quality and survival, warranting preventive measures to regulate the cellular functions that orchestrate tumor progression, therapy resistance, metastasis, and tumor relapse/recurrence. Globally, active efforts are underway to identify novel chemopreventive agents, define their mechanism(s) of action, and assess their clinical benefit. Some chemoprevention strategies (e.g., retinoids, difluoromethylornithine) have already been adopted clinically as part of maintenance phase therapy. Several agents are in the pipeline, while many others are in preclinical characterization. Here we review the classes of chemopreventive agents investigated for neuroblastoma, including cellular events targeted, mode(s) of action, and the level of development. Our review: (i) highlights the pressing need for new and improved chemopreventive strategies for progressive neuroblastoma; (ii) lists the emerging classes of chemopreventive agents for neuroblastoma; and (iii) recognizes the relevance of targeting dynamically evolving hallmark functions of tumor evolution (e.g., survival, differentiation, lineage transformation). With recent gains in the understanding of tumor evolution processes and preclinical and clinical efforts, it is our strong opinion that effective chemopreventive strategies for aggressive neuroblastoma are a near reality.

## INTRODUCTION

Neuroblastoma is the most common and deadliest cancer in infants. The challenge of curing neuroblastoma remains, as half of the children present with high-risk aggressive disease, which does not respond to current intensive multimodal clinical therapy. Although prevention of neuroblastoma genesis is not possible, prevention measures to delay disease progression are crucial for the survival of patients with therapy-defying high-risk neuroblastoma. Thus, global efforts are focused on identifying lead chemopreventive deliverables and developing new and improved chemopreventive strategies for high-risk neuroblastoma. Here we review the chemopreventive drugs (or strategies) identified, in the pipeline, and/or in clinical use for neuroblastoma.

Neuroblastoma is an extracranial solid tumor that originates from the precursor cells of the sympathetic nervous system during development. It is the most common cancer in infancy and accounts for about 10% of all childhood cancer deaths worldwide. During embryogenesis, the accumulation of several mutations and molecular rearrangements in sympathoadrenal lineage-committed neural crest cells drives neuroblastoma development^[1]^. Only 1%−2% of neuroblastoma is inherited (familial neuroblastoma) with a genetic predisposition in a locus mapped to the short arm of Chr16; familial neuroblastoma presents with multifocal primary tumors. The etiology of neuroblastoma genesis in the remaining 98%−99% remains unclear. Probable risk factors include the existence of other neurocristopathies (e.g., Hirschsprung’s disease, Klippel-Feil syndrome, Waardenburg’s syndrome, Ondine’s curse, Beckwith-Wiedemann syndrome, Cushing’s syndrome); fetal alcohol syndrome; maternal medications for seizure disorders (e.g., phenylhydantoin); products causing high blood pressure, sweating, headache, and abnormal heartbeats in the mother; and birth defects (congenital anomalies). It is generally understood that the genetic mutations that cause birth defects have a high likelihood of abnormality in cell lineage and the development of neuroblastoma. Unlike many adult cancers, in which their genesis can be linked to lifestyle variables (e.g., smoking, environmental exposure), no such risk factors have been identified in neuroblastoma.

Neuroblastomas can be stratified into classes based on their clinical behavior, namely clinically favorable disease and unfavorable high-risk disease. Patients who present with favorable neuroblastoma (about 40%), with near-triploid karyotypes, rare structural rearrangements, and expression of TrkA receptors, display a complete cure through spontaneous regression or maturation. Conversely, patients with unfavorable highrisk neuroblastoma, characterized by structural changes (deletions in 1p, 11q; gain in 17q) and expression of TrkB, display advanced disease stages with poor prognosis. More than half of high-risk patients show disease relapse with hematogenous metastasis and frequent relapses, with a rapidly decreasing survival timeline despite intensive multimodal clinical therapy. Clinical therapy for high-risk neuroblastoma comprises: (1) induction phase chemotherapy (cyclophosphamide, cisplatin/carboplatin, vincristine, doxorubicin, etoposide, topotecan) and load reduction surgery; (2) consolidation phase chemotherapy (carboplatin, etoposide, topotecan, busulfan and melphalan, thiotepa), radiotherapy (EBRT, MIBG RT) and stem cell transplant (autologous bone marrow transplantation [ABMT], peripheral blood stem cell reinfusion); and (3) maintenance phase treatment with retinoid drugs (13-cis-retinoic acid, isotretinoin), immunotherapy (dinutuximab), and immune-activating cytokine (GM-CSF, IL-2) treatment. Considering neuroblastoma heterogeneity and drug resistance, achieving a cure after the relapse of high-risk neuroblastoma is challenging. It is clear that the rapid reduction in the timeline between successive recurrences is due to the ongoing acquisition of genetic rearrangements in undifferentiated/poorly differentiated cancer. Hence, it is critical to identify new and effective chemoprevention strategies to delay or prevent these acquired events and/or lineage transformation in neuroblastoma cells and thereby defy tumor evolution into progressive disease.

**Figure 1.**
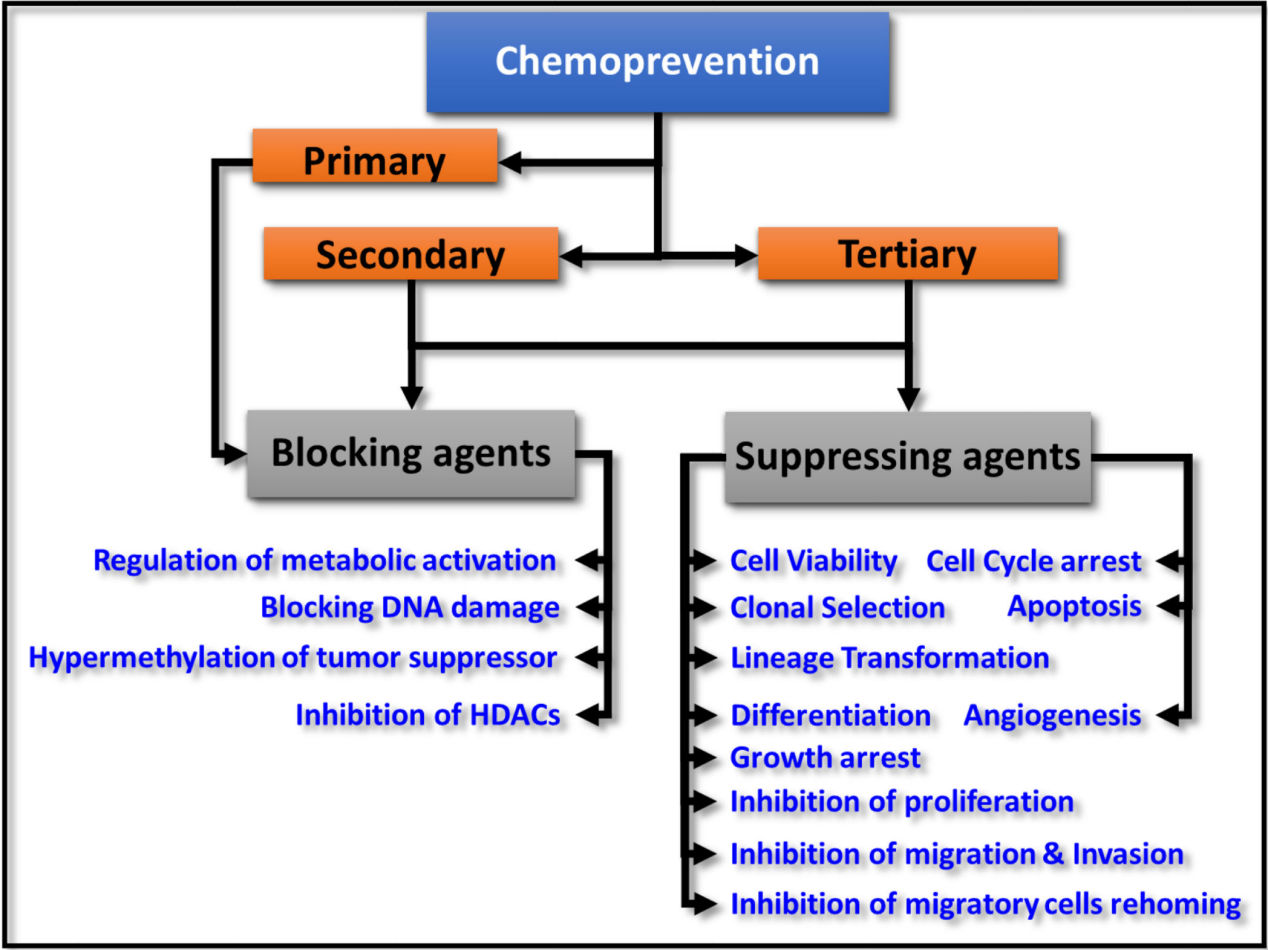
Schema showing the classification of chemopreventive strategies and the specific cellular functions targeted by each class of chemopreventive agent.

Chemoprevention is purposing/repurposing the natural, synthetic, or biological compounds to reverse, prevent, delay, or suppress tumor genesis, progression, and evolution. Based on the stage of tumor evolution, chemopreventive agents are graded as: (i) blocking agents (primary chemopreventive), the compounds that are used to block cancer initiation; and (ii) suppressing agents (secondary, the transition of a tumor from benign to malignant phenotype and tertiary, reduction of the risk of tumor recurrence following therapy), which reduce or delay tumor progression [Figure 1]. The modes of action for blocking agents include regulation of metabolic activation, blocking DNA damage [e.g., reactive oxygen species (ROS)], hypermethylation of tumor suppressors, and inhibition of histone deacetylases. Suppressing agents act by targeting the signaling pathways that drive cancer cell metabolism, cell proliferation, apoptosis, angiogenesis, lineage transformation, differentiation, clonal selection, and cancer stem cell stemness maintenance^[1,2]^. Apoptosis is arguably the prime cellular event for chemoprevention and has been extensively investigated (reviewed in detail elsewhere^[3]^). As with any chemotherapy drugs, the selection of chemopreventive agents is based on epidemiological data, human-mimicking preclinical investigations, and clinical trial outcomes. Although serious efforts are focused on identifying chemopreventive agents for deadly neuroblastoma, the clinical translation of these agents remains limited, principally attributed to the lack of a comprehensive and compiled understanding of the realized outcomes to date. Here we review the drugs investigated for chemoprevention of neuroblastoma, their strategies, the mechanism(s) of action, and outcomes. This review is focused on the chemopreventive agents that reduce, inhibit, or delay neuroblastoma progression.

## DISCUSSION

### Retinoids for neuroblastoma chemoprevention:

**Figure 2.**
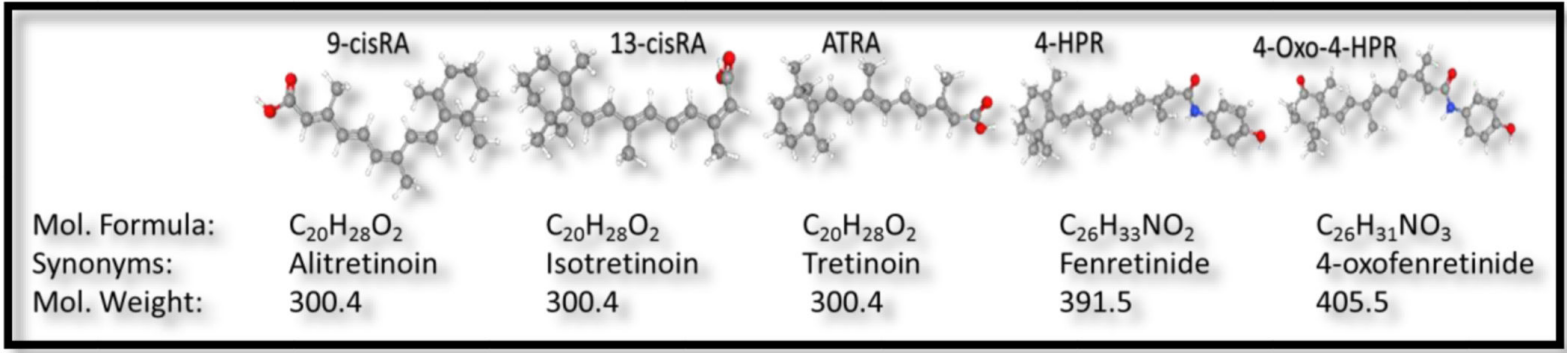
3D molecular structures, molecular formula and molecular weight of 9-cisRA, 13-ciRA, ATRA, 4-HPR, and 4-Oxo-4-HPR (a metabolite of 4-HPR) molecules. Images were adapted from PubChem^®^ (Available from: https://pubchem.ncbi.nlm.nih.gov/), National Library of Medicine, NIH.

**Figure 3.**
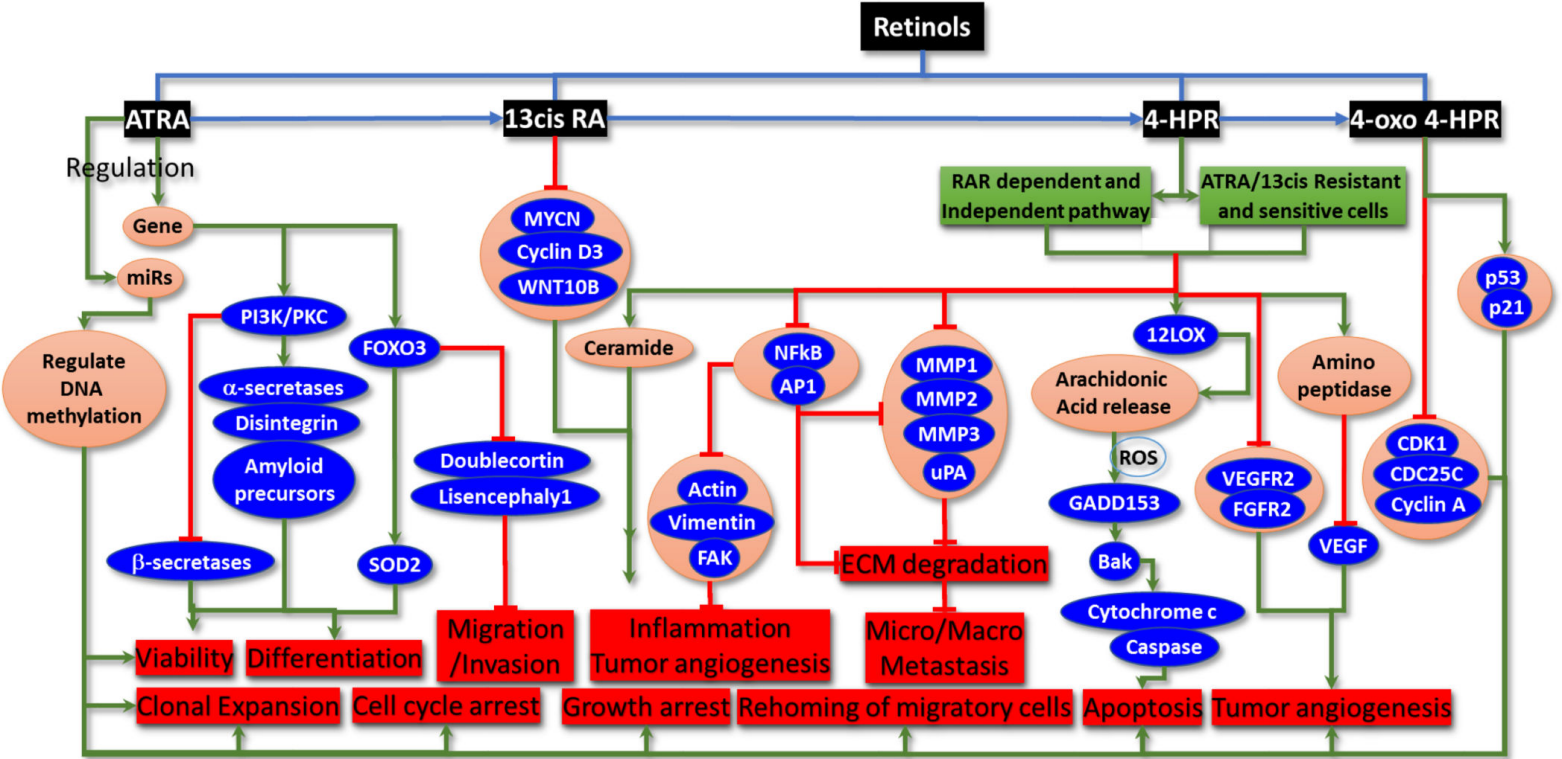
Simplified pathway map showing targeted signaling events altered or rearranged with retinoid treatment, their mechanism(s) of action, and the effect on key cellular functions involved in neuroblastoma evolution. Significantly, retinoids-regulated signaling converges on targeting the signaling pathways that regulate the hallmarks of neuroblastoma progression.

Retinoids are one major class of chemopreventive agents investigated in neuroblastoma. These retinoids are natural (cod liver oil, eggs, dairy products, yellow-/ orange-colored fruits and vegetables) or synthetic derivatives of vitamin A (retinol). Retinoids bind and transactivate retinoic acid receptors (RARs and RXRs) and are known to play crucial roles in cellular proliferation, differentiation, and apoptosis. Importantly, since retinol controls epithelial differentiation, it is regarded as a cancer prevention agent and has demonstrated benefit and efficiency in cancer control. Many synthetic retinoids have been developed and investigated as cancer-preventive or therapeutic agents [Table 1]. In human neuroblastoma, retinoic acid (RA) remains the most potent differentiation inducer. In that regard, chemical structures [Figure 2], pharmacokinetic properties, and mechanisms of action of RA metabolites (and their derivatives) in vitro, in vivo and in clinical settings have unveiled the neuroblastoma differentiation benefit of RA (reviewed in detail elsewhere^[4–6]^). Here we highlight some key signaling and functional events caused by retinoid therapy in high-risk and/or progressive neuroblastoma [Figure 3].

Several studies reported the benefit of all-trans-RA (ATRA) in preventing neuroblastoma pathogenesis. Redfern and colleagues reviewed the differential effects of 9-cis-RA and ATRA in neuroblastoma differentiation, gene expression, and inhibition of proliferation, as well as the mechanisms of action^[7]^. The involvement of microRNAs (miRs) in ATRA-driven chemoprevention is extensively documented (reviewed in detail elsewhere^[8]^). ATRA treatment resulted in neuroblastoma cell differentiation by selectively rearranging miRs and consequently altering the pathways of neuronal differentiation and maturation. Importantly, the molecular effects of ATRA treatment are not only rapid and robust but also sustained for a considerable period. In contrast, parallel studies showed that ATRA affects apoptosis or decreases neuroblastoma cell proliferation, rather than differentiation. Furthermore, it has been shown that ATRA contributed to the regulation of neuroblastoma cell migration and invasion, in addition to affecting differentiation. Although various mechanisms of ATRA function in neuroblastoma chemoprevention have been elucidated, it is particularly striking that ATRA regulates the miRs controlling DNA methylation, which, in turn, dictates decreased cell viability, clonal expansion, invasiveness, and rehoming of migratory cells, and contributes to their differentiation. In addition, ATRA has been shown to increase FoxO3a in neuroblastoma cells. FoxO3 is documented to drive cell cycle arrest, apoptosis, and growth arrest.

While promoting differentiation (SOD2 activation), RA also inhibits cell migration and invasion by decreasing doublecortin and lissencephaly-1. In addition, RA stimulates PI3K/PKC-dependent putative α-secretases, disintegrin metalloproteinases and amyloid precursor protein and regulates β-secretase cleavage in neuroblastoma cells. The nongenomic function of RA is mediated by RAR, activating PI3K/MAPK signaling-induced differentiation. The genomic function of ATRA involves CREB-phosphorylation-dependent caspase-8 transcription leading to neuroblastoma cell apoptosis.

The retinoid 13-cis-RA (13-cis-RA, isotretinoin), an isomer of ATRA, is a cancer chemoprevention drug that causes differentiation, decreased proliferation and inhibition of MYCN expression in progressive neuroblastoma cells. Consequently, isotretinoin serves as an adjunct treatment agent for neuroblastoma. Isotretinoin-mediated neuroblastoma cell differentiation is orchestrated by targeting MYCN, cyclin D3, and Wnt10B^[9]^. In a phase II study, 13-cis-RA showed modest activity against recurrent neuroblastoma^[10]^.

However, with the *in vitro* indications that 13-cis-RA-mediated growth arrest was sustained for weeks after removal, a high-dose pulse strategy (*vs*. continuous dosing) was considered in neuroblastoma patients. A phase I study of 13-cis-RA in high-risk neuroblastoma following ABMT using a high-dose intermittent schedule showed the ability to achieve effective doses, better tolerance, and an improved response rate. Consistent with this finding, a phase III trial in children with high-risk neuroblastoma who were progression-free after intensive or myeloablative chemoradiotherapy and ABMT, treated with high-dose intermittent 13-cis-RA showed significantly higher event-free survival (EFS)^[11]^. These studies affirm the significance of effective dosage (enough to sustain growth arrest), dosing strategy (intermittent), and time (immediately after chemotherapy) for increased benefits of 13-cis-RA treatment.

The major clinical limitations of retinoids are their toxicity and water insolubility. Thus, studies are focused on identifying retinoids that are well tolerated in humans with favorable outcomes in preneoplastic and neoplastic conditions. Despite the benefits of 13-cis-RA in improving the survival of high-risk neuroblastoma patients, the development of 13-cis-RA resistance is also a reality. Thus, global efforts focus on identifying better retinoids that lead to less or no resistance. In this regard, N-(4-hydroxyphenyl)-retinamide (4-HPR, fenretinide), a synthetic retinoid, has been shown to cause apoptosis and differentiation^[12]^, even in ATRA- and 13-cis-RA-resistant neuroblastoma cells. One of the mechanisms through which 4-HPR exerts neuroblastoma prevention is orchestrated apoptosis^[13–16]^. It has been shown that 4-HPR inflicts both RAR-dependent and -independent apoptosis. Mechanistically, 4-HPR-induced ROS leads to increased 12-lipoxygenase (12-LOX) activity that, in turn, oxygenates its substrate arachidonic acid, released from membrane phospholipids. These events result in the accumulation of ROS and ROS-dependent induction of growth and DNA damage (GADD)-inducible transcription factor GADD153^[17]^. Activation of GADD153 prompts the induction of BAK, and together they drive the cascade of events dictating the mitochondrial release of cytochrome c and activation of caspases, leading to apoptosis. Further, studies show that free radical generation could be one of the many mechanisms that drive 4-HPR-induced apoptosis and concluded that the effector pathway of 4-HPR-induced apoptosis is caspase-dependent, at least for neuroblastoma^[18]^. However, the 4-HPR-inflicted alterations in mitochondrial membrane potential and the resulting apoptosis are debatable^[18,19]^. Nevertheless, amphiphilic dextrins complexed with 4-HPR revealed improved bioavailability and *in vitro* cytotoxic efficacy in neuroblastoma cells^[20]^. 4-HPR bound to polyvinyl alcohol displayed improved anti-neuroblastoma tumor efficacy^[21]^. Treatment with 4-HPR encapsulated into nanoliposomes tagged with NGR peptides (NGR-NL[HPR]) that target the tumor endothelial cell marker aminopeptidase resulted in reduced tumor progression, inhibited tumor angiogenesis, and increased survival rates in a xenograft mouse model of neuroblastoma^[22]^. Functionally, NGR-NL[HPR] treatment resulted in the regulation of VEGF and matrix metalloproteinases (MMP2, MMP9). A phase I trial with higher doses of oral 4-HPR showed no systemic toxicity. Mechanistically, 4-HPR has been shown to elevate levels of ceramide in neuroblastoma cells^[14]^. A phase II study of oral 4-HPR in children with recurrent, refractory high-risk neuroblastoma showed prolonged stable disease but indicated the requirement for novel formulations to improve bioavailability [Table 1]^[23]^. In this regard, 4-HPR delivered orally in a powdered lipid complex displayed higher plasma levels, minimal toxicity, and excellent anti-tumor activity compared with other formulations^[24]^. Further, Villani and colleagues showed that 4-oxo-4-HPR, a metabolite of 4-HPR, regulated neuroblastoma cell proliferation^[25]^. 4-oxo-4-HPR caused the accumulation of cells in the G2-M phase by selectively regulating CDK1, cdc25c, and cyclin A, and promoted apoptosis through the activation of p53 and p21^[25]^. One of the critical clinical benefits of 4-HPR is its targeting of angiogenesis (angioprevention).

Anti-angiogenesis is a key cellular function of retinol that could serve as a tool for chemoprevention. The unique neoformation of blood vessels (angiogenesis) with leaky cell-cell connections crucially facilitates tumor progression and evolution. Anti-angiogenic strategies aim to arrest tumor growth through nutrient deprivation and enhance the delivery of therapeutic drugs. With the recent growth in anti-angiogenic strategies in cancer control, 4-HPR is recognized as a secondary cancer chemoprevention agent with antiangiogenic properties^[26]^. The angiopreventive activity of 4-HPR is through direct targeting of endothelial cell (EC) functions or by altering the production, release, and activation of growth factors and other determinants of angiogenic processes. For instance, 4-HPR inhibited EC growth by targeting VEGFR2 and FGFR2 and thereby prevented neuroblastoma biopsy-induced angiogenesis in the chick chorioallantoic membrane^[27,28]^. Studies have reported the benefit of retinoids in preventing cancer invasion and metastasis by targeting proteases (MMP-1, MMP-2, MMP3, uPA) involved in the degradation of the extracellular matrix (ECM). GD2-targeted immunoliposomes loaded with 4-HPR inhibited both the micro- and macrometastasis of neuroblastoma *in vivo*^[15]^. Other studies indicated that 4-HPR could effectively target upstream regulators of ECM degradation (AP1, NFκB), cell motility and adhesion (actin, vimentin, FAK), inflammation, and angiogenesis.

Overall, RA treatment in high doses can arrest neuroblastoma cell growth and induce morphological differentiation *in vitro*, and its inclusion after intensive chemotherapy and ABMT improves EFS in high-risk neuroblastoma. The biological functions of RA, including cellular differentiation and growth arrest, are mediated through RARs and RXR, the steroid/thyroid hormone family of transcription factors that possess distinct DNA- and RA-binding domains. These RARs and RXRs are highly expressed in neuroblastoma, and their expression levels correlate with sensitivity to RAs. The realized impact of RA in the prevention of neuroblastoma cell lineage transformation opened a new arena investigating several novel RA derivatives. In particular, the 9-cis-RA derivatives Ro13–6307 and 9-cis-UAB30 displayed promising benefits both in vitro and in preclinical in vivo settings. Likewise, many lead candidates, including RA-triazolyl compounds [i.e., (2E,4E,6E,8E)-(1-(2-nitrophenyl)-1H-1,2,3-triazol-4-yl)methyl, 3,7-dimethyl-9-(2,6,6-trimethylcyclohex-1-en-1-yl)nona-2,4,6,8-tetraenoate, and (2E,4E,6E,8E)-(1-(2-fluorophenyl)-1H-1,2,3-triazol-4-yl)methyl 3,7-dimethyl-9-(2,6,6-trimethylcyclohex-1-en-1-yl)deca-2,4,6,8-tetraenoate], and cyclic retinoic geranylgeranoic acid AT-R-retinoyl β-glucuronide (RAG) are being actively investigated in neuroblastoma settings. Additionally, it is noteworthy that a new class of RA derivatives [i.e., N-(4-hydroxyphenyl) amido (4HPTTNPB), and 4-hydroxybenzyl (4HBTTNPB)] that provide measurable benefits in other cancer settings and are in the line to be investigated in neuroblastoma. Interestingly, the studies were undertaken to understand the RAs and their derivatives unveil their potential to target stemness maintenance transcription machinery (NANOG, SOX2 and OCT3/4)-dependent lineage transformation of neuroblastoma cells, in addition to their established tumor regression and tumor cell differentiation effects. Although the major limitation of RAs and their derivatives has been their toxicity profile, promising new evidence from appropriate translatable preclinical models and proper dosing and combination strategies indicates the potential for rapid realization of pro-differentiation RA treatment for NB.

### Difluoromethylornithine for neuroblastoma chemoprevention:

The chemopreventive agent difluoromethylornithine (DFMO) has been widely studied in neuroblastoma evolution control. DFMO irreversibly inhibits ornithine decarboxylase (ODC), the rate-limiting enzyme of polyamine biosynthesis. High expression of ODC has been shown to drive neoplastic transformation in neuroblastoma. The significance and mechanism of polyamine metabolism in neuroblastoma and the role of MYCN in polyamine-regulated neuroblastoma progression have been reviewed in detail elsewhere^[29]^. DFMO-dependent inhibition of ODC has been shown to prevent disease progression in a preclinical setting. Importantly, DFMO targets LIN28/Let7 signaling and thereby affects the neuroblastoma cancer stem cell lineage^[30,31]^. DFMO has been under continuous clinical investigation for neuroblastoma since the first Phase 1 study that established a safe dose of 1,500 mg/m^2^ in relapsed patients. A Phase II study demonstrated clinically significant improvement in the survival of high-risk neuroblastoma patients with DFMO administration after standard COG therapy when compared with the no-DFMO matched controls^[31]^. Such a benefit of EFS maintenance with DFMO in neuroblastoma indicates its probable potential to prevent relapse in patients with high-risk disease [Table 1]. Consistent with that report, a subset analysis of a phase II trial evaluating DFMO as maintenance therapy to prevent relapse following standard or salvage therapy showed that DMFO is safe and associated with high survival for high-risk neuroblastoma^[32]^. With these promising and measurable outcomes, DFMO treatment could not only complement current maintenance therapy but also provide long-term benefits, particularly for children presenting with relapsed or primary refractory disease. With these proven benefits, one could speculate that the combination of DFMO with induction chemotherapy may produce sustainable tumor remission and increased patient survival.

### Other candidates:

A recent study utilizing a connectivity map approach with metastatic disease transcriptome data identified a synthetic drug, calcipotriol, a vitamin D3 analog, that could selectively target metastatic cells while sparing parental cells^[33]^. Calcipotriol, while reducing neuroblastoma cell proliferation and survival through vitamin D receptor signaling, also regulates cell migration by inducing RASSF_2_-dependent muting of the Hippo signaling pathway and Hippo pathway effectors (e.g., YAP and TAZ)^[33]^. With such a selective response and clear molecular driver, further studies could lead to the repurposing of calcipotriol as a chemopreventive agent for neuroblastoma. Similarly, noscapine, a known anti-microtubule agent, increases and activates p53, decreases cell survival, and prompts neuroblastoma cell death^[34]^.

#### Phytochemicals in the pipeline for neuroblastoma chemoprevention

**Figure 4.**
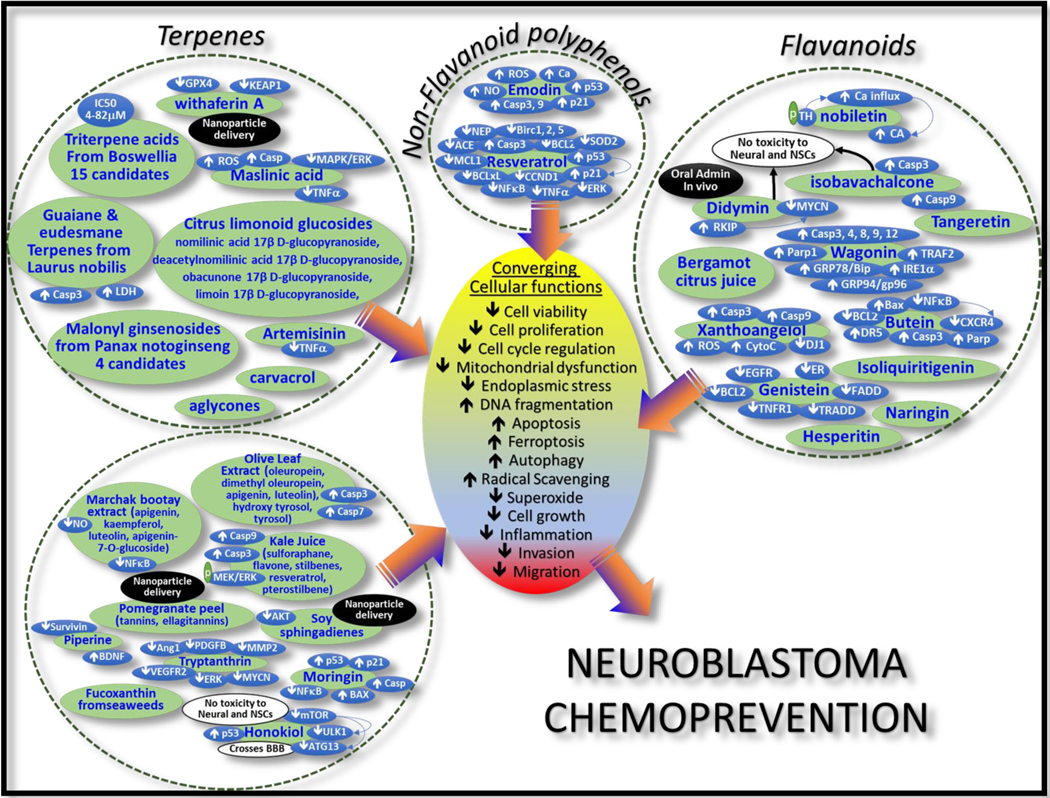
Cartoon showing diverse classes of phytochemicals investigated in vitro and in vivo for neuroblastoma chemoprevention, their mode of action and targeted cellular functions that dictate prevention of tumor progression, metastasis and disease evolution. Chemopreventive drugs target targets and/or signaling pathways, but largely the mode of action converge in defined sets of cellular/biological functions and conceptually supports the chemopreventive cocktail strategy for the development of effective molecular targeted maintenance therapy for neuroblastoma. Independent approaches displayed the systemic delivery feasibility, selective effect on tumor cells while sparing normal neural cells and neural stem cells, and excellent chemopreventive benefit of phytochemicals in the preclinical models of neuroblastoma.

A wide array of phytochemicals, including curcumin, resveratrol, quercetin, dihydroartemisinin, procyanidin, folate and folic acid, lycopene, anthocyanins, silibinin, sulforaphane, indole-3-carbinol, ellagic acid, genistein, and diallyl disulfide, have been extensively investigated for their benefits as chemopreventive agents in tumors, including neuroblastoma^[35]^. Here we discuss a few of these agents that have direct chemopreventive properties in neuroblastoma [Figure 4]. For ease and clarity, we restrict this review to only the sources of the phytochemicals, outcomes, cellular functions targeted, and genetic determinants involved.

### Terpene:

The cancer chemopreventive and cytotoxicity benefits of a panel of 15 lead candidate triterpene acids extracted from the resin of *Boswellia* trees were documented *in vitro* utilizing three neuroblastoma (IMR-32, SK-N-SH and NB-39) cell lines^[36]^. These compounds displayed strong prevention against chemical carcinogenesis, with the IC50 values ranging from 4.1–82.4 M. These triterpene acids could potentially benefit the prevention of neuroblastoma evolution [Figure 4]. The ethyl acetate fraction rich in guaiane and eudesmane terpenes from leaves of Mediterranean *Laurus nobilis* induced neuroblastoma cell apoptosis and thus represents a potent chemoprevention drug^[37]^. In neuroblastoma cells, this agent inhibited cell viability, increased LDH release and caspase 3 activation, and increased DNA fragmentation and radical scavenging capacity. Another monoterpene, carvacrol (CVC), induced a substantial decrease in cell proliferation without any significant DNA damage^[38]^, while the terpene and natural lactone, withaferin A, exerts canonical ferroptosis by inhibiting GPX4 activity, and non-canonical ferroptosis by targeting KEAP1 in neuroblastoma^[39]^. Such a bidirectional effect is highly effective in killing high-risk neuroblastoma cells *in vitro* and suppressing neuroblastoma growth *in vivo* when compared with chemotherapy drugs, including etoposide and cisplatin. Moving forward, this study utilized a withaferin A-encapsulated nanoparticle strategy for systemic delivery and clinical translation^[39]^. Another recent study identified and structurally characterized four new malonyl ginsenosides (the triterpene saponins) from the fresh fruits of the popular Chinese medicinal plant *Panax notoginseng*. Assessing the neuroblastoma cytotoxicity with these four new compounds and two known analogues, this study identified three lead candidates for neuroblastoma chemoprevention^[40]^.

Citrus limonoid glucosides from the class of furan-containing triterpenes, which differ in chemical structure from flavonoids, showed promising bioactivity against neuroblastoma [Figure 4]. For instance, four purified limonoid glucosides, limo in 17β D-glucopyranoside, obacunone 17β D-glucopyranoside, nomilinic acid 17β D-glucopyranoside, and deacetylnomilinic acid 17β D-glucopyranoside, displayed superoxide quenching activity, cell growth inhibition, and rapid apoptosis^[41]^. The same group also showed the relative beneficial effects of aglycones compared with glucosides in neuroblastoma prevention^[42]^. Artemisinin, a sesquiterpene lactone isolated from the aerial parts of *Artemisia annua*, was investigated for chemopreventive properties in neuroblastoma. The ethanolic extract of artemisinin cause significant anti-inflammatory and cytotoxic effects with the regulation of TNFα transcription^[43]^. Maslinic acid, a pentacyclic terpene found abundantly in hawthorn berries and olive fruit skins, is known for its pharmacological safety and anti-tumor, antidiabetogenic, antioxidant, antiviral, and anti-inflammatory properties, and exerted a remarkable neuroblastoma chemopreventive potential. Further, maslinic acid amplified ROS production, induced caspase-dependent apoptosis, and reduced neuroblastoma cell migration and invasion by selectively targeting the MAPK/ERK signaling pathway^[44]^.

### Flavonoids:

One class of chemopreventive agents that is extensively investigated is citrus flavonoids [Figure 4]. The citrus flavonoids (ethanolic extracts of citrus peels, tangeretin, nobiletin, hesperidin, didymin, hesperitin, and naringin) have numerous effects, including: high antioxidant potential, suppression of carcinogenesis, cell cycle regulation, programmed cell death, angioprevention, and antimetastasis. Nobiletin, a polymethoxy flavone, enhances catecholamine synthesis through the phosphorylation of tyrosine hydroxylase, driving calcium influx and catecholamine secretion^[45]^. Tangeretin and 5-demethyl nobiletin showed growth inhibitory effects in neuroblastoma with increased caspase3 activity and apoptosis^[46]^. Didymin exhibited p53 status-independent neuroblastoma cell killing *in vitro*. Importantly, oral administration of didymin significantly regressed neuroblastoma *in vivo*, without any toxicity to non-malignant cells, neural tissues, and neural stem cells^[47]^. Mechanistically, didymin induces transactivation of RKIP, a Raf-inhibitory protein that attenuates MYCN activation. The flavonoid-rich bergamot citrus juice inhibited neuroblastoma cell proliferation, adhesion, and invasion *in vitro*, and reduced the number of lung metastases in a spontaneous metastatic neuroblastoma model^[48]^.

Another dietary antioxidant compound, isoliquiritigenin (ISLQ), is a chalcone-type flavonoid that has been shown to increase cellular ROS and exert cytotoxic effects on MYCN-amplified neuroblastoma cells^[49]^. Importantly, ISLQ potentiated cisplatin-induced cytotoxicity and has been recognized as a potential deliverable as an adjunct therapy for high-risk neuroblastoma. Genistein, a natural isoflavone from soybeans, inhibits cancer cell growth by targeting protein tyrosine kinases and growth receptors like EGFR and ER. In neuroblastoma, studies have shown that genistein downregulates survival proteins (e.g., BCL2) and induces death factors and death domains (e.g., TNFR-1, TRADD, FADD)^[50]^. In our studies, we show that genistein serves as a lead second-stage chemoprevention agent by selectively targeting therapy pressure (radiotherapy)-driven response (unpublished data). Similarly, wogonin, a natural flavone isolated from the roots of Chinese skullcap, is a multitargeting chemoprevention agent that has been extensively investigated^[51]^. Wagonin regulates a number of key signaling pathways, including cell cycle arrest, autophagy, apoptosis, proliferation, invasion, and migration pathways. In neuroblastoma, it has been shown to increase mitochondrial dysfunction and endoplasmic reticulum stress by stimulating caspases (caspase-3, 4, 8, 9, 12), PARP1, GRP-78/Bip, GRP-94/gp96, IRE1α, and TRAF2^[52]^.

Butein, another flavonoid isolated from the stem bark of cashews, exhibited antioxidant, anti-inflammatory, and anti-tumor activities. Mechanistically, butein is known to: (i) regulate BCL2/Bax ratio, induce caspase activity, and consequently drive apoptosis; (ii) stimulate DR5 and mediate caspase-3-dependent apoptosis in TRAIL-resistant cells; and (iii) inhibit NFκB and dictate NFκB-mediated CXCR4-dependent tumor cell migration. In neuroblastoma, butein treatment resulted in a significant ROS increase and cell cycle arrest, regulated the Bcl-2/Bax ratio, stimulated caspase and PARP activity, decreased cell viability, and induced apoptosis^[53]^.

Chalcones, an important class of plant flavonoids from edible fruits and vegetables, have antioxidant, antimicrobial, anti-inflammatory, anticancer, cytotoxic, and immunosuppressive bioactivities^[54]^. Xanthoangelol, a major chalcone from the stem of *Angelica keiskei*, can induce caspase-3-dependent apoptosis in neuroblastoma cells^[55]^. Functionally, xanthoangelol-triggered ROS activate apoptosis by releasing cytochrome c and activating caspase-9 in both drug-resistant and drug-sensitive neuroblastoma cells. Importantly, the xanthoangelol-induced oxidative stress regulates DJ-1 protein, thereby contributing to the loss of antioxidant function and acceleration of apoptosis^[55]^. In another study, Nishimura and colleagues compared the neuroblastoma chemopreventive potential of eight different chalcones^[56]^; while all chalcones displayed significant cytotoxicity against neuroblastoma cells, the lead compound isobavachalcone was safe for normal cells even at high concentrations, but induced caspase-3/9-dependent apoptosis in neuroblastoma cells.

### Non-flavonoid polyphenols:

Several other dietary supplements have been repurposed for neuroblastoma chemoprevention [Figure 4]. Resveratrol, a dietary polyphenol with documented anticancer, anti-aging, anti-inflammatory, antimicrobial, and neuroprotective capabilities, has been investigated as a chemopreventive agent in neuroblastoma. Resveratrol caused neuroblastoma cell cytotoxicity with S-phase arrest, decreased clonal expansion, and increased mitochondria-mediated intrinsic caspase-dependent apoptosis by selectively targeting cyclin D1, Bcl-2, Bcl-xL, and Mcl-1 proteins. Studies also show that resveratrol suppressed neuroblastoma growth *in vivo*^[57]^, and that the resveratrol-mediated chemopreventive effects (decrease in cell viability, cell cycle arrest, and increase in cell death) are orchestrated by increased p53 expression and nuclear translocation that drives activation of p21 and Bax^[58]^. Our studies indicated that resveratrol could benefit the prevention of radio-resistance by targeting radiotherapy-associated NFκB-dependent transcriptional machinery, attenuating eNos, Erk1/2, SOD2, Akt1/2/3, p50, p65, pIκBα, TNFα, and Birc-1, −2, −5, and inducing apoptosis^[59]^. Prolonged exposure of neuroblastoma cells to resveratrol has been shown to activate neutral endopeptidase and angiotensin-converting enzymes, inhibiting cell proliferation and promoting differentiation^[60]^.

Emodin, one of the prime polyphenol constituents of rhubarb root, is widely used as an immunosuppressive, anti-inflammatory, anti-atherosclerotic, and vasorelaxant agent. It has been shown to exert anticancer properties in various tumor types, including breast cancer, hepatoma, leukemia, lung cancer, and neuroblastoma. Emodin is an anthraquinone that stimulates apoptosis through ROS-mediated mitochondria-dependent pathways. In neuroblastoma, emodin induces apoptosis by concurrently increasing ROS, cytoplasmic-free Ca, and NO, leading to the loss of mitochondrial membrane potential and activation of caspases 9 and 3, p53, and p21, and thus inflicting cell death^[61]^.

### Others:

Olive leaf extract caused a time- and dose-dependent inhibition of cell proliferation, arrest in cell cycle progression, accumulation of cells in the sub-G0 phase, upregulation of caspases 3 and 7, and increased apoptosis in 2D and 3D neuroblastoma models^[62]^. Olive leaves are known to possess elevated levels of secoiridoids (oleuropein, dimethyl oleuropein), flavonoids (apigenin and luteolin), and other phenols (hydroxytyrosol, tyrosol). In an interesting *in vitro* transepithelial anti-neuroblastoma model, kale juice induced ROS and S phase arrest and inhibited neuroblastoma cell growth while also increasing the transepithelial electrical resistance^[63]^. Although rich in flavones/flavonoids, kale juice also contains sulforaphane, flavone, stilbenes, resveratrol, and pterostilbene. Sulforaphane (SFN), an isothiocyanate present in cruciferous vegetables, is known to suppress neuroblastoma cell viability, inhibit DNA synthesis, activate caspase, and prompt apoptosis by targeting autophagy^[64]^. Another study investigating the chemopreventive potential of sulforaphane in neuroblastoma indicated that sulforaphane treatment resulted in the depletion of mitochondrial membrane potential, increased caspases 9 and 3, and heightened phosphorylation of MEK/ERK (without generating ROS) and cell killing^[65]^.

Soy-derived sphingolipids, sphingadienes, are also cytotoxic to neuroblastoma cells^[66]^. Mechanistically, sphingadienes inhibit AKT phosphorylation and orchestrate caspase-dependent apoptosis and autophagy. The chemopreventive effect of sphingadienes was further demonstrated by decreased neuroblastoma growth and loss of AKT activation when nanoparticle-loaded sphingadienes were systemically administered^[66]^.

A recent investigation of moringin [isothiocyanate 4-(α-L-rhamnopyranosyloxy) benzyl C] in neuroblastoma demonstrated a significant chemopreventive potential by targeting cell growth and activation of cell death^[67]^. Moringin is an isothiocyanate from *Moringa oleifera*, described as a miracle tree for its multiple parts (seeds, flowers, roots, leaves, bark) and multiple uses (functional food, medicine).

Traditional medicine uses it in the management of many diseases, and it has anti-inflammatory, antimicrobial, and cancer chemopreventive properties. In neuroblastoma, moringin induced cell cycle arrest at the G2 and S phases, transactivated and translated p53, p21, and Bax, regulated NFκB, and promoted intrinsic apoptotic cascade (caspases transactivation and cleavage)^[67]^.

The medicinal plant Marchak bootay is commonly used as a tonic in traditional medicine. A Marchak bootay extract that is rich in saturated fatty acids and flavonoids (apigenin, kaempferol, luteolin, apigenin-7-O-glucoside) showed excellent pharmacological safety *in vivo.* Ahmad and colleagues iterated the benefits and targets of various fractions of marchak in the context of NFκB inhibition, NO activity, and antiproliferative activity in neuroblastoma cells^[68]^.

Honokiol, a biphenolic small molecule isolated from the bark and leaves of magnolia, is used in traditional medicine as an anxiolytic, antithrombotic, anti-depressant, anti-emetic, and antimicrobial. In neuroblastoma, honokiol is known to induce cell cycle arrest, DNA fragmentation, and apoptosis through the activation of p53^[69]^. Importantly, honokiol efficiently traverses the blood-brain barrier and can kill neuroblastoma cells while sparing normal neuronal cells^[70]^. In addition, honokiol treatment induced autophagy through multifarious signaling mechanisms in neuroblastoma cells^[71,72]^. Furthermore, honokiol treatment resulted in the regulation of mTOR phosphorylation, leading to activation of protein kinases (ULK1, ATG13) and consequent suppression of autophagy^[71]^.

Rich in tannins, ellagitannins, and anthocyanins, including ellagic acid, pomegranate peel has been shown to exhibit antimicrobial, anticancer, anti-obesity, antidiabetic, antiulcerogenic, and antihypertensive proprieties. A study assessing the enhanced bioactivity of pomegranate peel extract adsorbed in calcium carbonate nanocrystals showed that nano-formulations resulted in high radical scavenging ability and better antiproliferative activity^[73]^.

Another important class of promising chemopreventive agents is the phytochemicals isolated from marine seaweeds. For instance, the abundant marine xanthophyll fucoxanthin is the key pigment that generates the brown color in brown seaweeds. This carotenoid is documented to possess anti-oxidative and anticancer cell properties. Many studies showed that fucoxanthin can inhibit cancer cell growth and induce apoptosis in multifarious tumor settings, including human neuroblastoma cells^[74,75]^.

Alkaloids have also been exploited as chemopreventive agents in neuroblastoma. Tryptanthrin, an indoloqinazoline alkaloid isolated from many natural sources, displayed antimicrobial, anti-inflammatory, anti-protozoan, and anti-parasitic activity. In neuroblastoma cells, tryptanthrin is shown to inhibit MYCN expression and thereby potentiate cell killing^[76]^. Importantly, the same group demonstrated the antiangiogenic activities of tryptanthrin both *in vitro* and *in vivo*. Tryptanthrin inhibited endothelial cell proliferation, migration, tube formation, and angiogenesis in mice. Mechanistically, tryptanthrin regulates pro-angiogenic Ang-1, PDGFB, MMP2, and VEGFR2-mediated ERK1/2 signaling in ECs^[77]^. Another dietary alkaloid extracted from the fruits and roots of pepper, piperine, has been used in Indian traditional medicine for the treatment of gastrointestinal and respiratory diseases. Piperine exhibits unparalleled bioactivities, including anti-inflammatory, antimetastatic, anticancer, larvicidal, leishmanicidal, immunosuppressive, antimycobacterial, and antiparasitic activities. In neuroblastoma, piperine has been shown to inhibit survivin, a crucial survival driver^[78]^. Further, alkamides from white pepper were shown to increase endogenous BDNF protein and consequently promoted RA-induced neurite outgrowth^[79]^.

Traditionally, various phytochemicals are used in combination to prevent many chronic diseases, including cancer. In general, chemoprevention, particularly secondary chemo/radio prevention of neuroblastoma using phytochemicals is promising based on both efficacy and well-documented safety profiles. Herein, we describe the chemopreventive activities of numerous phytochemicals in neuroblastoma and iterate the cellular signaling pathways that mediate their specific anti-neuroblastoma effects. Evidently, these phytochemicals target critical signaling pathways involved in the development and evolution of neuroblastoma, and could aid in developing multi-targeted therapies. The traditional medicine practices of the east Asian region highlight the potential benefits of a combinatorial strategy in the chemoprevention of cancer, including neuroblastoma^[35]^. Although the combination approach is not in clinics at present, such a strategy is evolving in laboratory and preclinical studies^[80]^. Research is focused on defining and characterizing active compounds, understanding synergy, and identifying plausible analogs and combinations that cause complete cancer remission. Preliminary outcomes demonstrate that the combination of various phytochemicals displays defined effects on multiple cellular functions (or targets) with additive or synergistic responses, has low dose requirements compared with stand-alone treatments, and also has low toxicity. More importantly, such synergistic or additive activity of phytochemicals could complement standard chemotherapy, leading to reduced dosing and/or frequency of chemotherapy drug use and thus minimizing toxicity.

Here we have listed chemopreventive phytocompounds that are subject to ongoing anticancer-related research - research that aims to define their chemopreventive activity and identify new therapeutic targets. The specific dietary compounds described in this review may constitute potent cancer chemopreventive agents, and the consumption of foods including such bioactive compounds has protective and therapeutic effects on various types of cancers. The polyphenolic compounds from plants have an immunomodulatory role that identifies and destroys cancer cells through anti-angiogenic effects. Chemopreventive drugs enhance chemotherapy and radiotherapy efficacies via multiple signal transduction pathways. Since oxidative stress plays a vital role in the pathogenesis of many cancers, the antioxidant effect of dietary phenolic compounds might act as a promising strategy to prevent cancer. Dietary antioxidant phytochemicals are abundant in plants and represent different classes of compounds with various mechanisms of action on tumors. Hence, chemoprevention through diets rich in plant-based antioxidants shows great potential in reducing the risk factors associated with cancer progression.

## CONCLUSION AND FUTURE PROSPECTIVES

**Figure 5.**
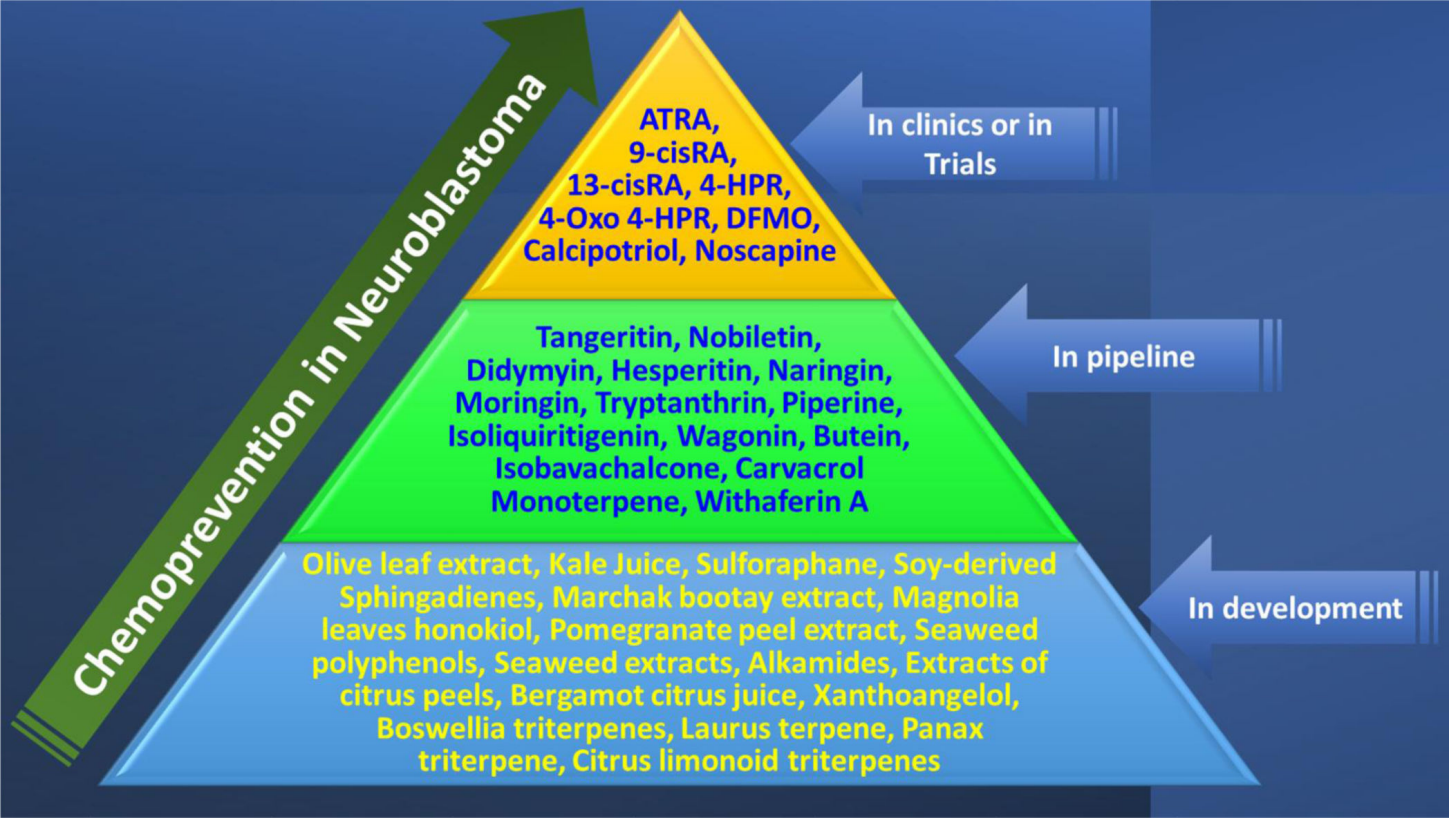
Pyramid showing the evolution profile of chemopreventive agents for neuroblastoma. While some retinoids and DFMO are used as part of maintenance therapy, some are still being characterized in clinical trials. A large group of lead candidates that show excellent chemopreventive properties are in the pipeline for clinical translation. In addition, a sizable group of potential candidates for neuroblastoma chemoprevention are currently being characterized.

Preventing the genesis, disease progression, therapy resistance, and evolution of progressive neuroblastoma is a critical need to save the lives of thousands of children afflicted with this deadly disease. Although complete prevention of neuroblastoma genesis is not feasible, secondary and tertiary chemoprevention is achievable and could effectively improve the quality of life and reduce childhood cancer deaths. Identifying an effective chemopreventive strategy is critical for high-risk neuroblastoma patients, particularly those presenting with aggressive metastatic disease and/or with therapy defying progressive disease. Accordingly, current global efforts are focused on developing clinically translatable chemopreventive strategies by selectively targeting the genetic determinants that drive neuroblastoma disease aggressivity and the molecular rearrangements that dictate therapy resistance [Figure 5]. Functionally, most preventive strategies that are currently in development, in the pipeline, or in trials are targeted towards differentiation, angioprevention, or lineage transformation prevention. Despite the ongoing colossal efforts, there are significant holdups in developing new and effective preventive strategies that intervene in multiple events of neuroblastoma evolution. The future of neuroblastoma chemoprevention lies in the ability to overcome the challenges, including but not limited to:

- The requirement of synergistic activity for multi-agent combination treatments.
- Better targeting of the neuroblastoma cells in the narrow window between de novo acquisition of molecular rearrangements and adaptation. The timeframe estimates of when chemoprevention should commence pose a significant barrier.
- Effective targeting of tumor cells while normal cells are selectively protected.
- Enhancing the effectiveness and/or decreasing the toxicity of first-line modalities i.e., surgical resection, radiation therapy and chemotherapy.
- Defining molecular strategies for selective targeting of neuroblastoma cells while sparing normal cells. Owing to the heterogeneity of the putative target expression in neuroblastoma cells, it is critical to identify a chemopreventive strategy (or cocktail) that is aimed at unifying target(s) in all tumor cells that are also absolutely lacking in normal cells.
- The requirement to identify those patients susceptible to developing disease progression, therapy resistance, and disease relapse. Likewise, it is critical to identify individuals who might show a positive/ negative response with select chemoprevention intervention.
- Requirement for the patient-, disease-, and combination-specific dose, dose-rate estimate of the chemopreventive agent.

Although the concept of chemoprevention is over four decades old, the evolution of chemopreventive strategies in neuroblastoma remains in its initial stages. Here, in this review, we cover the major and most promising agents investigated for neuroblastoma chemoprevention to date, indicating their mechanism(s) of action and outcomes. Although there may be other effective agents in the neuroblastoma setting, we focused entirely on agents covered in PubMed. While some of the agents reviewed here are currently at the validation stages, other compounds are extensively investigated and have been translated to clinics. For instance, retinoic acid treatment for neuroblastoma cell differentiation is well established and is currently included in the clinical standard of care. Likewise, the promising benefits of the ornithine decarboxylase inhibitor DFMO have been recognized, and it is in the pipeline for maintenance therapy for neuroblastoma. In the footprints of this success to date, there is an opportunity to identify new and improved chemopreventive strategies for select subsets of aggressive, metastatic, or progressive neuroblastoma. In this review, we have listed the variety of agents studied specifically for secondary and tertiary chemoprevention in neuroblastoma and the signaling pathways or molecular targets selectively involved. Most of these agents converge in targeting hallmarks of neuroblastoma evolution, including active cell replication, dedifferentiation, stemness maintenance, cell growth, invasion, migration, inflammation, angiogenesis, mitochondrial dysfunction, endoplasmic stress, autophagy and therapy resistance. Even though there are variations in the regulation of signaling pathways, the encouraging outcomes from independent studies, in general, indicate the possibility of a chemopreventive agents cocktail strategy for the development of effective molecularly targeted maintenance therapy for neuroblastoma [Figure 4]. It is pertinent to mention that neuroblastoma arises from neural crest stem cells during early embryonic development; the signaling pathways that orchestrate disease evolution are complex and often opposite to those involved in tumors of epithelial origin. Hence, repurposing effective chemopreventive agents that perform against tumors of epithelial origin may have limitations in neuroblastoma, in turn demanding the development and characterization of unique targeting approaches. It is apparent that in-depth research is needed with a detailed understanding of the performance of any agent (or combination of agents) addressing the challenges listed above to develop effective chemopreventive strategies for this deadly disease.

In conclusion, an effective chemopreventive strategy is a requisite if we are to successfully treat therapy-defying progressive neuroblastoma. The success of an effective chemoprevention strategy could immensely contribute to lowering disease relapse and associated mortality. Identification of potential agents coupled with recognition of risk factors will pave the way for better management of disease progression. It is encouraging that many agents are in clinical trials and many more are in the pipeline for neuroblastoma. However, it is critical to consider and unveil sufficient experimental evidence regarding the safety, clearance and off-target physiognomies of the agents prior to clinical trials and translation.

## Figures and Tables

**Table 1.** List of clinical trials that investigated the inclusion of chemoprevention strategies in neuroblastoma

Title of the Study	Intervention	Sponsor/Collaborators	Phase	Clinical Trials Identifier
**Preventative Trial of Difluoromethylornithine (DFMO) in High-Risk Patients with Neuroblastoma That is in Remission**	DFMO	Wake Forest University Health Sciences …,	Phase 2	NCT02395666
**N2012–01: Phase 1 Study of Difluoromethylornithine (DFMO) and Celecoxib With Cyclophosphamide/Topotecan**	DFMO	NANT, NCI	Phase 1	NCT02030964
**An Intermediate Expanded Use Trial of DFMO**	DFMO	Giselle SaulnierSholler..,	Phase 1|Phase 2	NCT03581240
**Safety Study for Refractory or Relapsed Neuroblastoma with DFMO Alone and in Combination with Etoposide**	DFMO	Wake Forest University Health Sciences …,	Phase 1	NCT01059071
**Neuroblastoma Maintenance Therapy Trial**	DFMO	Wake Forest University Health Sciences …,	Phase 2	NCT02679144
**Eflornithine (DFMO) and Etoposide for Relapsed/Refractory Neuroblastoma**	DFMO	Wake Forest University Health Sciences …,	Phase 2	NCT04301843
**Pediatric Precision Laboratory Advanced Neuroblastoma Therapy**	DFMO	Wake Forest University Health Sciences …,	Phase 2	NCT02559778
**Study of DFMO in Combination with Bortezomib for Relapsed or Refractory Neuroblastoma**	DFMO	Giselle SaulnierSholler..,	Phase 1|Phase 2	NCT02139397
**A Phase II Trial of All-Trans-Retinoic Acid in Combination With Interferon-Alpha 2a in Children With Recurrent Neuroblastoma or Wilms’ Tumor**	ATRA	National Cancer Institute (NCI)..,	Phase 2	NCT00001509
**Expanded Access Study of Fenretinide Lym-X-Sorb Plus Ketoconazole in Neuroblastoma**	Fenretinide	South Plains Oncology Consortium	Phase 1|Phase 2	NCT02075177
**N2004–03: Intravenous Fenretinide in Treating Young Patients with Recurrent or Resistant Neuroblastoma**	Fenretinide	National Cancer Institute (NCI)..,	Phase 1	NCT00646230
**Fenretinide Lym-X-Sorb + Ketoconazole + Vincristine for Recurrent or Resistant Neuroblastoma**	Fenretinide	South Plains Oncology Consortium..,	Phase 1	NCT02163356
**N2004–04: Fenretinide LXS in Treating Patients With Recurrent, Refractory, or Persistent Neuroblastoma**	Fenretinide	National Cancer Institute (NCI)..,	Phase 1	NCT00295919
**Fenretinide in Treating Children with Recurrent or Resistant Neuroblastoma**	Fenretinide	National Cancer Institute (NCI)	Phase 2	NCT00053326
**Fenretinide in Treating Children with Solid Tumors**	Fenretinide	National Cancer Institute (NCI)	Phase 1	NCT00003191
**Vorinostat With or Without Isotretinoin in Treating Young Patients with Recurrent or Refractory Solid Tumors, Lymphoma, or Leukemia**	Fenretinide	National Cancer Institute (NCI)	Phase 1	NCT00217412
**Neuroblastoma Protocol 2008: Therapy for Children with Advanced Stage High Risk Neuroblastoma**	Isotretinoin	St. Jude Children’s Research Hospital	Phase 2	NCT00808899
**Naxitamab and Granulocyte-Macrophage Colony Stimulating Factor (GMCSF) and Isotretinoin for Consolidation of Patients With High-Risk Neuroblastoma in First Remission.**	Isotretinoin	Y-mAbs Therapeutics	Phase 2	NCT04909515
**Isotretinoin in Treating Young Patients With High-Risk Neuroblastoma**	Isotretinoin	National Cancer Institute (NCI)	Not Applicable	NCT00939965
**Therapy for Children With Advanced Stage Neuroblastoma**	Isotretinoin	St. Jude Children’s Research Hospital ‥,	Phase 2	NCT01857934
**A Study of the Effect of Hu3F8/GM-CSF Immunotherapy Plus Isotretinoin in Patients in First Remission of High-Risk Neuroblastoma**	Isotretinoin	Memorial Sloan Kettering Cancer Center..,	Phase 2	NCT03033303
**Anti-GD2 3F8 Monoclonal Antibody and GM-CSF for High-Risk Neuroblastoma**	Isotretinoin	Memorial Sloan Kettering Cancer Center	Not Applicable	NCT02100930
**Response-based Treatment of High-risk Neuroblastoma**	Isotretinoin	Samsung Medical Center	Phase 2	NCT02771743
**Combination Chemotherapy and Surgery with or Without Isotretinoin in Treating Young Patients With Neuroblastoma**	Isotretinoin	COG National Cancer Institute (NCI)	Phase 3	NCT00499616
**Combination Chemotherapy Followed by Stem Cell Transplant and Isotretinoin in Treating Young Patients With High-Risk Neuroblastoma**	Isotretinoin	National Cancer Institute (NCI)	Not Applicable	NCT00526318
**Imetelstat Given Intravenously Alone and With Standard 13-Cis-Retinoic Acid in Children with Neuroblastoma**	Isotretinoin	NCIC Clinical Trials Group..,	Phase 1	NCT01916187
**High Dose Chemotherapy and Autologous Transplant for Neuroblastoma**	Isotretinoin	University of Minnesota..,	Not Applicable	NCT01526603
**ch14.18 Pharmacokinetic Study in High-risk Neuroblastoma**	Isotretinoin	United Therapeutics	Phase 1|Phase 2	NCT01592045
**Monoclonal Antibody Therapy Plus Etoposide in Treating Patients With Neuroblastoma**	Isotretinoin	Memorial Sloan Kettering Cancer Center, NCI	Phase 2	NCT00004110
**Combination Chemotherapy With or Without Filgrastim Before Surgery, High-Dose Chemotherapy, and Radiation Therapy Followed by Isotretinoin With or Without Monoclonal Antibody in Treating Patients With Neuroblastoma**	Isotretinoin	University of Leicester, NCI	Phase 3	NCT00030719
**Observation, Combination Chemotherapy, Radiation Therapy, and/or Autologous Stem Cell Transplant in Treating Young Patients With Neuroblastoma**	Isotretinoin	Gesellschaft fur Padiatrische Onkologie..,	Phase 3	NCT00410631
**ZD6474 Alone and in Combination with Retinoic Acid in Pediatric Neuroblastoma**	Isotretinoin	M.D. Anderson Cancer Center..,	Phase 1	NCT00533169
**Combination Chemotherapy Plus Peripheral Stem Cell Transplantation in Treating Children with Newly Diagnosed Neuroblastoma**	Isotretinoin	COG National Cancer Institute (NCI)	Phase 2	NCT00017368
**Biological Therapy in Treating Patients With Neuroblastoma That Has Not Responded to Previous Treatment**	Isotretinoin	Memorial Sloan Kettering Cancer Center..,	Phase 2	NCT00089258
**3F8/GM-CSF Immunotherapy Plus 13-Cis-Retinoic Acid for Primary Refractory Neuroblastoma in Bone Marrow**	Isotretinoin	Memorial Sloan Kettering Cancer Center	Phase 2	NCT01183897
**Multiple Therapies in Treating Patients with Advanced Neuroblastoma**	Isotretinoin	Memorial Sloan Kettering Cancer Center//.	Phase 2	NCT00040872
**3F8/GM-CSF Immunotherapy Plus 13-Cis-Retinoic Acid for Consolidation of Second or Greater Remission of High-Risk Neuroblastoma**	Isotretinoin	Memorial Sloan Kettering Cancer Center	Phase 2	NCT01183884
**3F8/GM-CSF Immunotherapy Plus 13-Cis-Retinoic Acid for Consolidation of First Remission After Non-Myeloablative Therapy in Patients with High-Risk Neuroblastoma**	Isotretinoin	Memorial Sloan Kettering Cancer Center	Phase 2	NCT01183429
**A Study of MAb-3F8 Plus Granulocyte-Macrophage Colony-Stimulating Factor (GM-CSF) Versus 13-cis-Retinoic Acid (RA) Plus GM-CSF in Primary Refractory Neuroblastoma Patients**	Isotretinoin	United Therapeutics	Phase 2	NCT00969722
**Therapy for Children with Neuroblastoma**	Isotretinoin	St. Jude Children’s Research Hospital..,	Phase 2	NCT00135135
**Combination Chemotherapy and Peripheral Stem Cell Transplantation in Treating Patients with Neuroblastoma**	Isotretinoin	COG National Cancer Institute (NCI)	Phase 3	NCT00004188
**Induction Chemotherapy Using Cyclophosphamide and Topotecan in Treating Patients Who Are Undergoing Autologous Peripheral Stem Cell Transplantation for Newly Diagnosed or Progressive Neuroblastoma**	Isotretinoin	COG National Cancer Institute (NCI)	Phase 1	NCT00070200
**Biological Therapy, Sargramostim, and Isotretinoin in Treating Patients with Relapsed or Refractory Neuroblastoma**	Isotretinoin	COG National Cancer Institute (NCI)	Phase 2	NCT01334515
**Lenalidomide and Dinutuximab With or Without Isotretinoin in Treating Younger Patients With Refractory or Recurrent Neuroblastoma**	Isotretinoin	National Cancer Institute (NCI)	Phase 1	NCT01711554
**Monoclonal Antibody Ch14.18, Sargramostim, Aldesleukin, and Isotretinoin After Autologous Stem Cell Transplant in Treating Patients with Neuroblastoma**	Isotretinoin	National Cancer Institute (NCI)	Phase 3	NCT01041638
**NB2013-HR German (GPOH) / Dutch (DCOG) Trial**	Isotretinoin	University of Cologne	Phase 2	NCT02641782
**Aflac ST1001 Prolonged Isotretinoin**	Isotretinoin	Emory University.,	Phase 1	NCT01319838
**90Y DOTA/Retinoic Acid for Neuroblastoma and Neuroendocrine Tumor (NET)**	Isotretinoin	University of Iowa..,	Phase 2	NCT01048086
**Long Term Continuous Infusion ch14.18/CHO Plus s.c. Aldesleukin (IL-2)**	Isotretinoin	University Medicine Greifswald..,	Phase 1|Phase 2	NCT01701479
**Treatment With Dinutuximab, Sargramostim (GM-CSF), and Isotretinoin in Combination With Irinotecan and Temozolomide After Intensive Therapy for People With High-Risk Neuroblastoma (NBL)**	Isotretinoin	COG National Cancer Institute (NCI)	Phase 2	NCT04385277
**Dinutuximab, Sargramostim, and Combination Chemotherapy in Treating Patients with Newly Diagnosed High-Risk Neuroblastoma Undergoing Stem Cell Transplant**	Isotretinoin	National Cancer Institute (NCI)	Phase 2	NCT03786783
**Monoclonal Antibody Therapy With Sargramostim and Interleukin-2 in Treating Children With Neuroblastoma**	Isotretinoin	National Cancer Institute (NCI)	Phase 1	NCT00005576
**High-Dose 3F8/GM-CSF Immunotherapy Plus 13-Cis-Retinoic Acid for Consolidation of First Remission After Myeloablative Therapy and Autologous Stem-Cell Transplantation**	Isotretinoin	Memorial Sloan Kettering Cancer Center	Phase 2	NCT01183416
**Vorinostat and Isotretinoin in Treating Patients with High-Risk Refractory or Recurrent Neuroblastoma**	Isotretinoin	National Cancer Institute (NCI)	Phase 1	NCT01208454
**Oral Liquid 13-cis-retinoic Acid (13-CRA)**	Isotretinoin	Nova Laboratories Limited	Phase 1|Phase 2	NCT03291080
**Comparing Two Different Myeloablation Therapies in Treating Young Patients Who Are Undergoing a Stem Cell Transplant for High-Risk Neuroblastoma**	Isotretinoin	COG National Cancer Institute (NCI)	Phase 3	NCT00567567
**Isotretinoin With or Without Dinutuximab, Aldesleukin, and Sargramostim Following Stem Cell Transplant in Treating Patients with Neuroblastoma**	Isotretinoin	National Cancer Institute (NCI)	Phase 3	NCT00026312
**Iobenguane I-131 or Crizotinib and Standard Therapy in Treating Younger Patients With Newly-Diagnosed High-Risk Neuroblastoma or Ganglioneuroblastoma**	Isotretinoin	COG National Cancer Institute (NCI)	Phase 3	NCT03126916
**Induction Therapy Including 131 I-MIBG and Chemotherapy in Treating Patients with Newly Diagnosed High-Risk Neuroblastoma Undergoing Stem Cell Transplant, Radiation Therapy, and Maintenance Therapy With Isotretinoin**	Isotretinoin	COG National Cancer Institute (NCI)	Not Applicable	NCT01175356
